# Association of Initial Low Serum Selenium Level with Infectious Complications and 30-Day Mortality in Multiple Trauma Patients

**DOI:** 10.3390/nu11081844

**Published:** 2019-08-09

**Authors:** Soon Bo Choi, Yun Tae Jung, Jae Gil Lee

**Affiliations:** Department of Surgery, Yonsei University College of Medicine, Seoul 03722, Korea

**Keywords:** multiple trauma, infectious complications, selenium deficiency

## Abstract

Low serum selenium levels are commonly observed in critically injured multiple trauma patients. This study aimed to identify the association between initial serum selenium levels and in-hospital infectious complications in multiple trauma patients. We retrospectively reviewed multiple trauma patients admitted between January 2015 and November 2017. We selected 135 patients whose serum selenium levels were checked within 48 h of admission. Selenium deficiency was defined as a serum selenium level <70 ng/mL. Survival analyses of selenium deficiency and 30-day mortality were performed. Multivariate logistic regression analysis was performed to identify the association between initial serum selenium level and in-hospital infectious complications. Thirty-day mortality (8.3% vs. 0.0%; *p* = 0.018) and incidence rates of pneumonia (66.7% vs. 28.3%; *p* < 0.001) and infectious complications (83.3% vs. 46.5%; *p* < 0.001) were higher in patients with selenium deficiency than in patients without selenium deficiency. Kaplan–Meier survival cures also showed similar results (log rank test, *p* = 0.021). Of 135 patients, 76 (56.3%) experienced at least one infectious complication during admission. High injury severity score (ISS, odds ratio (OR) 1.065, 95% confidence interval (CI) 1.024–1.108; *p* = 0.002) and selenium deficiency (OR 3.995, 95% CI 1.430–11.156; *p* = 0.008) increased the risk of in-hospital infectious complications in multiple trauma patients. Patients with selenium deficiency showed higher 30-day mortality and higher risks of pneumonia and infectious complications.

## 1. Introduction

Traumatic injury is one of the most common causes of death worldwide. More than 5 million people die due to traumatic injuries every year [1]. The first peak in the trimodal model of trauma mortality includes deaths occurring at the scene of the traumatic event. In such cases, death occurs within minutes of severe and fatal injuries. The second peak in the model includes early deaths, which occur hours after arrival at the hospital [2]. The proportion of deaths in these two peaks has not changed over time, because these deaths depend mainly on the mechanism and severity of the damage caused by an irreversible fatal injury to a patient even before they obtain any medical assistance. By contrast, the proportion of deaths in the third peak in the trimodal model, known as late deaths that occur days to weeks after injury, has dropped dramatically over time [3]. Although hemorrhage and brain injury are common causes of death in both the early and late peaks of the trimodal model [4], infection and sepsis following multiorgan failure has become one of the major causes of death in the late phases of trauma [3,4,5,6]. This condition is induced by an initial cascade of inflammation resulting in multiple organ damage, which is then aggravated by sepsis, caused by increased susceptibility of the patient [7]. Numerous changes and advancements have occurred in sepsis treatment strategies over time, and these improvements have contributed to the reduction in late mortality [8]. Nevertheless, many aspects of this condition are still unknown or unclear, and further research may lead to improvement in patient outcomes.

The role of trace element levels in sepsis treatment has not yet been clearly defined. Selenium, one of the important trace elements, regulates immunity and inflammation in the body. This property of selenium in improving immune function has beneficial effects on disease course of autoimmune thyroiditis [9]. Furthermore, the antioxidative property of selenium plays an important role not only in immune response and inflammation but also in various aspects in human health, especially in cancer chemoprevention and heart disease [10,11].

Selenium deficiency has been known to negatively affect immune cells during activation, differentiation, and proliferation [11]. Low serum selenium levels, with increased oxidative stress and inflammatory biomarkers, are often observed in critically ill patients; moreover, low serum selenium levels have been associated with adverse clinical outcomes [11,12]. Therefore, we hypothesized that trauma patients with low serum selenium levels may develop infectious complications at a higher frequency and, therefore, have a higher risk of adverse outcomes. Thus, in this study, we aimed to identify the association between initial serum selenium levels and in-hospital infectious complications in multiple trauma patients.

## 2. Materials and Methods

### 2.1. Study Population

We retrospectively reviewed the electronic medical records (EMRs) of 1083 trauma patients who were admitted to our university hospital between January 2015 and December 2017. Of these, we selected 135 patients whose serum selenium levels were measured within 2 days of admission.

### 2.2. Data Collection

We collected data of baseline characteristics of the patients, such as age, sex, injury severity score (ISS), revised trauma score (RTS), and trauma and injury severity score (TRISS). TRISS comprises a weighted combination of age, ISS, and RTS to predict probability of survival of injured patients after trauma. It is the most commonly used tool since its introduction in 1983 [13]. These scores are importantly used in trauma patients to evaluate their severity and predict the outcomes. Data of serum selenium levels and clinical outcomes, including 30-day mortality and development of pneumonia or other infectious complications, were also recorded. These data were obtained from EMRs. Patients were classified into two groups according to their serum selenium levels, with a cut-off value of 70 ng/mL; baseline characteristics and clinical outcomes were compared between these two groups. Patients were also classified into two groups according to the occurrence of infectious complications.

This study was approved by the Institutional Review Board (IRB No. 4-2019-0482), and the informed consent was waived due to the retrospective nature of the study. 

### 2.3. Statistical Analysis

All results are presented as average ± standard deviation or frequency (%), as appropriate. The Student’s *t*-test was used for comparison of two continuous variables. The chi-square test or Fisher’s exact test was used to compare frequencies. Kaplan–Meier survival curves were plotted for survival analysis of the two groups, and the log rank test was used to compare these survival curves. Multivariate logistic regression analysis was performed to identify the risk factors for infectious complications, and their odds ratios (ORs) were calculated. The findings were considered to be statistically significant when *p* values were <0.05. Statistical analysis was performed using SPSS^®^ Statistics 25.0 (IBM Corp., Armonk, NY, USA).

## 3. Results

### 3.1. Baseline Characteristics

Among 135 patients, 36 had selenium deficiency, whereas 99 did not. Patients with selenium deficiency had lower RTS (6.352 ± 2.026 vs. 7.470 ± 1.114; *p* = 0.003), higher ISS (26.44 ± 13.64 vs. 18.07 ± 11.36; *p* = 0.002), and lower TRISS (72.17 ± 32.06 vs. 90.58 ± 19.14; *p* = 0.002) than patients without selenium deficiency. The proportion of patients requiring vasopressors within 24 h of admission (22.2% vs. 4.0%; *p* = 0.003) and that of patients requiring continuous renal replacement therapy (CRRT) (13.9% vs. 2.0%; *p* = 0.015) were higher in patients with selenium deficiency than in those without selenium deficiency. Frequency of transfusion within 24 h of admission was also high in patients with selenium deficiency (6.03 ± 8.06 packs vs. 0.74 ± 1.48 packs; *p* < 0.001) (Table 1).

### 3.2. Clinical Outcomes

**Figure 1:**
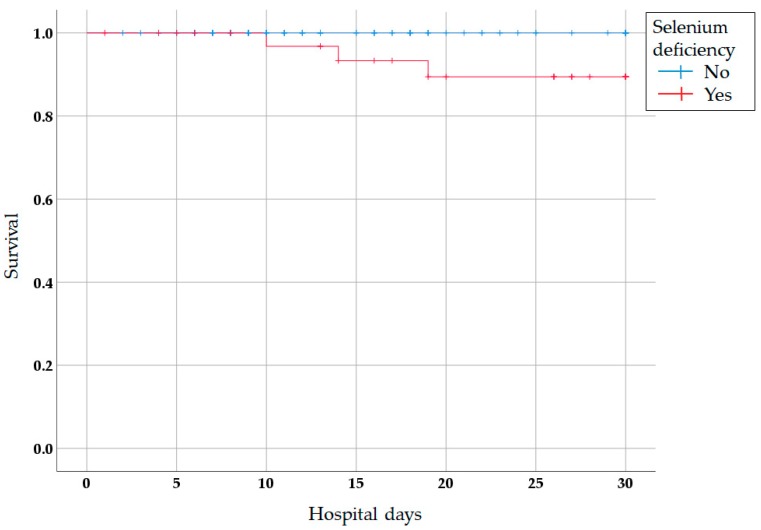
Kaplan–Meier survival curves for 30-day mortality versus selenium deficiency. The survival curves demonstrate lower predicted survival in patients with selenium deficiency than in those without selenium deficiency (*p* = 0.021).

Hospital length of stay (HLOS; 41.39 ± 53.55 vs. 24.31 ± 48.87 days; *p* = 0.083) and in-hospital mortality (8.3% vs. 1.0%; *p* = 0.058) showed no significant differences between the two groups. However, intensive care unit stay (12.69 ± 12.95 vs. 5.41 ± 8.39 days; *p* = 0.003) was longer and 30-day mortality (8.3% vs. 0.0%; *p* = 0.018) was higher in patients with selenium deficiency than in those without selenium deficiency. Newly developed pneumonia (66.7% vs. 28.3%; *p* < 0.001) and infectious complications (83.3% vs. 46.5%; *p* < 0.001) occurred more frequently in patients with selenium deficiency than in those without selenium deficiency (Table 2). The Kaplan–Meier survival curves also showed significant differences in 30-day mortality between the two groups (log rank test, *p* = 0.021) (Figure 1).

### 3.3. Risk Analysis for Occurrence of Infectious Complications

Patients who developed infectious complications had similar age (48.80 ± 18.77 vs. 48.15 ± 18.83; *p* = 0.842) and sex distribution (male (%)/female (%), 54 (71.1)/22 (28.9) vs. 45 (76.3)/14 (23.7); *p* = 0.496) with those who did not develop infectious complications. Patients who developed infectious complications had higher RTS (6.82 ± 1.89 vs. 7.61 ± 0.47; *p* = 0.001), ISS (24.32 ± 13.44 vs. 15.24 ± 9.09; *p* < 0.001), and TRISS (78.64 ± 29.54 vs. 94.74 ± 10.81; *p* < 0.001) than those who did not. Vasopressor use (14.5% vs. 1.7%; *p* = 0.012) and CRRT (9.2% vs. 0.0%; *p* = 0.018) was more common in patients who developed infectious complications than in those who did not (Table 3).

### 3.4. Multivariate Logistic Regression Analysis of Development of Infectious Complications

Multivariate logistic regression analysis showed that patients with higher ISS (OR 1.065, 95% confidence interval (CI) 1.024–1.108; *p* = 0.002) and patients with selenium deficiency (OR 3.995, CI 1.430–11.156; *p* = 0.008) had higher odds of developing infectious complications (Table 4).

## 4. Discussions

In our study, trauma patients with selenium deficiency had higher 30-day mortality and higher rates of developing pneumonia and infectious complications than trauma patients without selenium deficiency. The odds of developing infectious complications during hospital stay were higher in patients with high ISS and those with low serum selenium levels.

Consistent with our findings, many previous studies also reported an association between low initial selenium levels and adverse outcomes in critically ill patients [12,14,15,16]. However, the causal relationship was unclear in these studies. Low initial selenium levels may affect patients in terms of adverse outcomes, but contrarily, the severity of illness may have affected the selenium concentration. In our study, patients with selenium deficiency had higher initial ISS and TRISS as well as higher incidence of vasopressors use and CRRT, showing that low selenium levels are associated with disease severity in some way. However, this relationship cannot be clarified in retrospective studies.

Some randomized control studies have reported that supplementary selenium did not improve survival in critically ill patients [17,18,19]. However, in these studies, selenium supplementation was provided for a randomly selected study population, regardless of the patients’ individual serum selenium levels. Further studies are needed to investigate whether selenium supplementation has a positive effect on the survival of critically ill patients with selenium deficiency. Some studies have reported that adequate nutritional supplementation only benefited selected patients with high nutritional risks; adequate nutrition did not have positive effects on patients with low nutritional risks [20,21,22]. Another study showed that corticosteroid therapy only had a beneficial effect on septic shock patients with high inflammatory marker levels [23]. In this context, selenium supplementation could only be beneficial for patients with selenium deficiency.

Our study had certain limitations. This study had a retrospective design and involved a small study population; however, very few studies have evaluated the relation between low initial serum selenium levels and adverse outcomes in trauma patients. We were also unable to prove the causal relationship between initial low selenium levels and adverse outcomes; however, our study showed that low selenium levels could possibly result in adverse outcomes in trauma patients and that selenium supplementation could improve outcomes in these patients. In the future, randomized controlled trials are required to prove the positive effect of selenium supplementation on critically ill patients with selenium deficiency. This will not only clarify the causal relationship between low serum selenium levels and adverse outcomes but also change the ambiguous stance on selenium supplementation in the current guidelines.

## 5. Conclusions

Multiple trauma patients with higher ISS and selenium deficiency had higher risks for infectious complications. Patients with selenium deficiency had higher 30-day mortality and higher rates of developing pneumonia and infectious complications than patients without selenium deficiency.

## Figures and Tables

**Table 1:** Baseline characteristics.

Variables	Selenium Deficiency		*p* Value
	Yes (*n* = 36)	No (*n* = 99)	
Age, years	52.67 ± 18.50	47.01 ± 18.67	0.121
Sex, (M/F) (%)	27 (75.0)/9 (25.0)	72 (72.7)/27 (27.3)	0.792
RTS, *n*	6.352 ± 2.026	7.470 ± 1.114	0.003
ISS, *n*	26.44 ± 13.64	18.07 ± 11.36	0.002
TRISS, *n*	72.17 ± 32.06	90.58 ± 19.14	0.002
Selenium, ng/mL	59.23 ± 5.57	96.20 ± 24.67	<0.001
Vasopressor used within 24 h, *n* (%)	8 (22.2)	4 (4.0)	0.003
CRRT, *n* (%)	5 (13.9)	2 (2.0)	0.015
pRBC transfusion in 24 h, packs	6.03 ± 8.06	0.74 ± 1.48	<0.001

M/F = male/female, RTS = revised trauma score, ISS = injury severity score, TRISS = trauma and injury severity score, CRRT = continuous renal replacement therapy, pRBC = packed red blood cells.

**Table 2:** Clinical outcomes.

Variables	Selenium Deficiency		*p* Value
	Yes (*n* = 36)	No (*n* = 99)	
ICU stay, days	12.69 ± 12.95	5.41 ± 8.39	0.003
HLOS, days	41.39 ± 53.55	24.31 ± 48.87	0.083
In-hospital mortality, *n* (%)	3 (8.3)	1 (1.0)	0.058
30-day mortality, *n* (%)	3 (8.3)	0 (0.0)	0.018
Newly developed pneumonia, *n* (%)	24 (66.7)	28 (28.3)	<0.001
Infectious complications, *n* (%)	30 (83.3)	46 (46.5)	<0.001
Wound complications, *n* (%)	10 (27.8)	24 (24.2)	0.676

ICU = intensive care unit, HLOS = hospital length of stay.

**Table 3:** Baseline characteristics of patient groups according to occurrence of infectious complications.

Variables	Infectious Complications		*p* Value
	Yes (*n* = 76)	No (*n* = 59)	
RTS, *n*	6.82 ± 1.89	7.61 ± 0.47	0.001
ISS, *n*	24.32 ± 13.44	15.24 ± 9.09	<0.001
TRISS, *n*	78.64 ± 29.54	94.74 ± 10.81	<0.001
Selenium deficiency, *n* (%)	30 (39.5)	6 (10.2)	<0.001
Vasopressor used within 24 h, *n* (%)	11 (14.5)	1 (1.7)	0.012
CRRT, *n* (%)	7 (9.2)	0 (0.0)	0.018
pRBC transfusion within 24 h, packs	2.76 ± 5.58	1.36 ± 3.77	0.083

M/F = male/female, RTS = revised trauma score, ISS = injury severity score, TRISS = trauma and injury severity score, CRRT = continuous renal replacement therapy, pRBC = packed red blood cells.

**Table 4:** Multivariate logistic regression analysis.

Variables	Odds Ratio	95% CI	*p* Value
ISS	1.065	1.024–1.108	0.002
Selenium deficiency	3.995	1.430–11.156	0.008

ISS = injury severity score, CI = confidence interval.
